# Effects of Real-Time Visual Finger-Movement Feedback on Finger Tapping Performance in Older Adults

**DOI:** 10.3390/geriatrics11050145

**Published:** 2026-10-15

**Authors:** Shingo Takahashi, Yuki Kuroiwa, Noriko Sakurai, Daishi Takahashi, Naoki Kodama

**Affiliations:** 1 Department of Healthcare Informatics, Faculty of Health and Welfare, Takasaki University of Health and Welfare, 37-1 Nakaorui-machi, Takasaki 370-0033, Gunma, Japan; kuroiwa-y@takasaki-u.ac.jp (Y.K.); takahashi-d@takasaki-u.ac.jp (D.T.); 2 Department of Radiological Sciences, Faculty of Medical Science and Technology, Gunma Paz University, 1-7-1 Tonya-machi, Takasaki 370-0006, Gunma, Japan; n-sakurai@paz.ac.jp; 3 Department of Radiological Technology, Faculty of Medical Technology, Niigata University of Health and Welfare, 1398 Shimami-cho, Kita-ku, Niigata 950-3198, Niigata, Japan; kodama@nuhw.ac.jp

**Keywords:** finger mobility exercise, finger tapping performance, finger training, motor feedback system, older adult motor skills, sensorimotor training

## Abstract

**Background/Objectives:** Manual dexterity is closely associated with cognitive function and frailty among older adults, highlighting the importance of finger rehabilitation and broader sensorimotor training in older adults. This study investigated whether a real-time visual augmented feedback system for finger movements used during a finger-tapping task could enhance tapping performance in older adults. The study also aimed to examine whether feedback effects differ between the relatively simple simultaneous tapping task and the more demanding alternating tapping task. **Methods:** Participants included 37 middle-aged and older adults aged 60 years or older (7 men, 30 women). A magnetic sensor-based finger-tapping device was used to measure finger-tapping performance while participants received real-time feedback on their finger movements. Performance was evaluated using speed-related indices, including the number of taps, and coordination-related measures. **Results:** A significant Task × Feedback interaction in tap number was observed in the nondominant hand but not the dominant hand. Feedback increased tap number during alternating tapping in both hands, but not during simultaneous tapping; coordination measures were unchanged. **Conclusions:** The findings suggest that the real-time visual feedback system for finger movement may lead to immediate improvements in finger motor performance among older adults. They also suggest task-dependent feedback effects, with a significant Task × Feedback interaction observed only in the nondominant hand. Future studies should analyze differences based on movement type and investigate the persistence of these effects.

## 1. Introduction

Hand function in older adults can be impaired by musculoskeletal disorders such as osteoarthritis of the hand [1]. Globally, the number of patients with osteoarthritis is projected to increase even further in the future due to the growth of the aging population [2]. As people age, skeletal muscle function declines, resulting in a reduced ability to exert task-specific muscle strength or to precisely regulate muscle force during movement [3]. Finger function also declines with age [4]. Finger function is associated with activities of daily living (ADLs), such as housework [5], and training involving finger exercises has been shown to improve ADLs [6]. Recent studies have indicated that finger dexterity is associated with both frailty and cognitive function [7,8,9], highlighting the importance of maintaining and improving finger function in older age. Among these aspects of finger function, finger coordination plays an important role in performing daily activities [5].

Furthermore, aging is known to cause changes in motor speed, coordination, and timing accuracy in hand and finger movements [10]. Bilateral coordinated movements include in-phase (simultaneous) and anti-phase (alternating) patterns, which are supported by distinct neural control processes [11,12]. In particular, alternating movement tends to become unstable with aging, leading to increased variability and movement errors [13]. The precision and stability of movement depend on sensory feedback from visual and somatosensory inputs, and the processing of this information may be affected by aging [14,15]. Therefore, providing appropriate feedback in response to age-related declines in motor function may effectively improve motor performance.

Feedback is an effective method for improving motor performance [16], and learning ability is maintained even in older age [17]. Feedback can be classified as intrinsic or extrinsic, depending on whether it originates from the individual’s own sensory system or from external sources [18]. Extrinsic feedback, often referred to as augmented feedback, provides additional information regarding movement execution or outcomes and is widely used to facilitate motor learning [18,19]. However, the effectiveness of feedback may vary depending on task difficulty and participant characteristics. In relatively simple motor tasks requiring postural control, a low-frequency feedback group (67%) has been reported to perform better in retention tests compared with the 100% feedback group [20]. According to attentional focus theory, an external focus of attention is more effective for motor learning than an internal focus [21]. In older adults, directing attention externally toward motor effects is more effective for motor learning than directing attention internally toward one’s own movements [22]. Concurrent visual feedback has been reported to improve motor performance in older adults; for example, Yamamoto et al. displayed the target and actual grasping forces on a monitor during a force-adjustment task and observed improved performance between the pre-test and the 24 h retention test [23].

In this context, we developed a real-time visual augmented feedback system for finger movements that provides waveform-based visual information representing finger-tapping movements [24]. Repetitive bimanual tapping involves repeated opening and closing movements of the thumb and index fingers [25], together with timing control to maintain the required phase relationship between the two hands [26]. Unlike discrete feedback, continuous kinematic feedback can provide ongoing information about movement patterns. Continuous visual feedback can provide continuous temporal information about ongoing movement and support online movement control [27,28]. However, processing continuous visual information while performing bimanual movements may be demanding for older adults, given age-related differences in the use of visual and proprioceptive information [14,15] and evidence that age-related differences in bimanual coordination become more pronounced when effortful processing is required [13]. At the same time, older adults have been reported to rely more heavily on vision than proprioception when estimating hand position [15]. Thus, waveform-based feedback may facilitate online monitoring and correction of finger movements, while its continuous presentation may increase attentional demands. Accordingly, the net effect of waveform-based feedback on motor performance in older adults remains to be empirically determined.

In our previous study, this system improved finger-tapping performance among young adults [24]. However, because aging is accompanied by changes in motor control, sensorimotor integration, and movement coordination, it remains unclear whether older adults respond similarly to real-time visual feedback and whether the effectiveness of feedback differs according to movement complexity. To our knowledge, few studies have examined whether the immediate effects of real-time visual finger movement feedback vary across different finger-tapping tasks in older adults.

Therefore, this study examined (1) whether a real-time visual finger movement feedback system improves finger-tapping performance among older adults, and (2) whether its effects differ between simple and more demanding tapping tasks. The primary outcome was the number of taps, whereas all other finger-tapping parameters were analyzed as secondary outcomes. We hypothesized that real-time visual feedback would improve finger-tapping performance in older adults, with greater effects observed during the more demanding alternating tapping task.

## 2. Materials and Methods

### 2.1. Participants

The sample size was calculated using G*Power version 3.1.9.7. Based on the G*Power calculation (effect size: 0.5, statistical power: 0.8, α error probability: 0.05, difference between two dependent means (matched pairs)), the required sample size was 34. The study included middle-aged and older individuals aged 60 years or above who were recruited from the Tokyo Toy Museum. The sample comprised 37 individuals (7 men, 30 women; mean age: 71.3 ± 6.0 years; Mini-Mental State Examination score: 29.2 ± 1.3). Of these, 36 were right-handed, while one was left-handed. Two participants who had been retrained from left- to right-handedness were classified as right-handed. The exclusion criteria were suspected cognitive impairment, motor disorders, or impairments of the hands, arms, or fingers; however, no candidates were excluded. Participants performed finger-tapping tasks while receiving real-time feedback, and their performance was evaluated. Participants were briefed on the study before participation and provided written informed consent.

### 2.2. Finger Motor Feedback System

We evaluated finger performance during feedback conditions using a real-time visual finger motor feedback system developed by our group. The system configuration was identical to that described in our previous study [24] and consisted of a PC, a magnetic sensor-based finger-tapping device (UB-2; Maxell Holdings, Tokyo, Japan), and a feedback monitor. A Python (version 3.10.9) program continuously captured the region of the UB-2 measurement screen displaying the finger-movement waveforms and presented it on the feedback monitor. The displayed waveforms changed continuously in response to the participants’ ongoing finger movements, providing visual information on tapping speed and coordination between the two hands. The UB-2 sampled finger-movement signals at 100 Hz. In a technical evaluation conducted using the same computer and software configuration as in the experiment, the mean software-loop processing time across two runs of 1000 iterations each was 30.94 ± 2.11 ms, corresponding to an update rate of approximately 32.3 Hz. The processing loop included screen capture, image cropping, resizing, and display-event processing. The feedback monitor operated at a refresh rate of 60 Hz. These specifications enabled the finger-movement waveforms to be continuously updated and presented with low latency during task performance.

**Figure 1:**
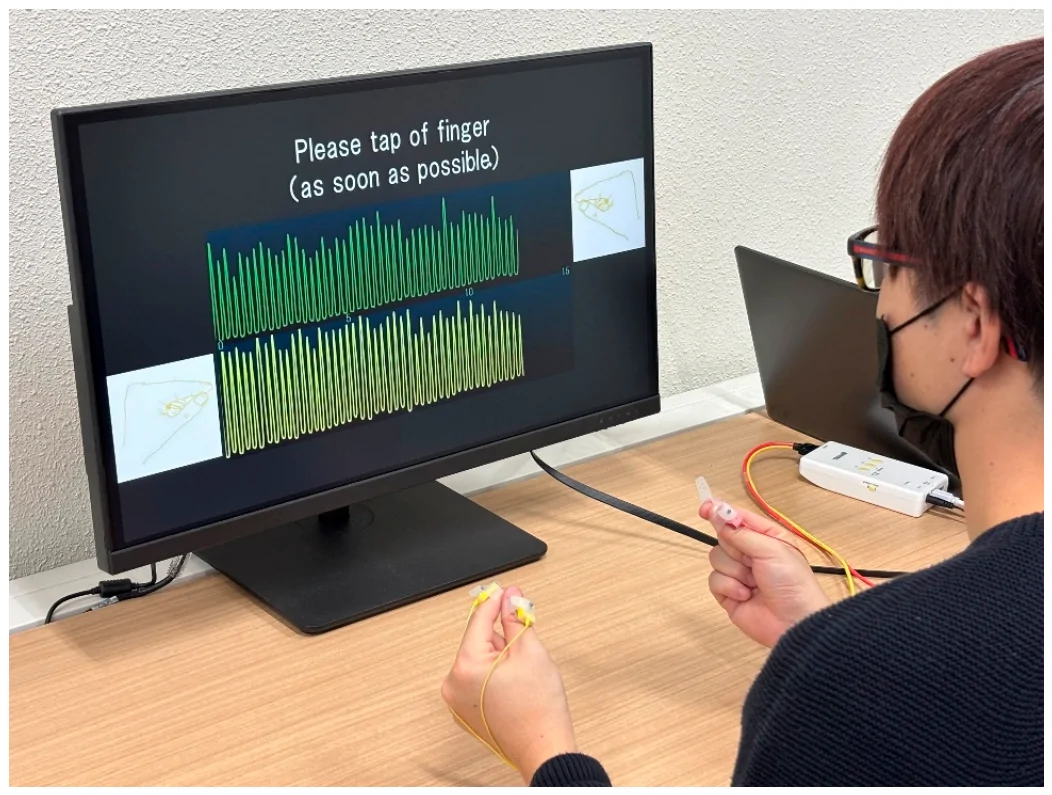
Photograph showing the experimental setup during feedback. The participant is wearing the UB-2, and their finger movements are displayed as waveforms on the monitor in front of them. The instructions on the feedback screen shown in the figure have been translated into English for illustrative purposes; the actual experiment used Japanese instructions.

The system was noninvasive and involved minimal restraint, making it suitable for use by older adults. Prior to the experiment, the system was explained to the participants, who were instructed to look at the feedback monitor during tapping and not at their hands. To maintain a natural task environment without imposing excessive constraints, the participants’ hands were not occluded. Figure 1 illustrates the configuration of the feedback system. Participants performed the task while seated at a desk, and measurements were conducted in a quiet indoor environment with minimal external stimulation. To maintain consistency with our previous study, we used a similar visual feedback design. In the no-feedback condition, a fixation cross was displayed at the center of a black screen on the same monitor used in the feedback condition to standardize gaze direction. Participants were instructed to maintain their gaze on the fixation cross and not to look at their hands.

### 2.3. Finger-Tapping Task

Finger tapping was performed as described in previous reports [25,29,30]. Tapping was performed using the thumb and index finger of both hands. Two movement types were assessed: simultaneous tapping, in which the fingers opened and closed simultaneously, and alternating tapping, in which the fingers of the left and right hands opened and closed alternately. Participants were instructed to tap with a target amplitude of 4 cm and as quickly as possible while maintaining accuracy. The 4 cm distance served as an instructional target rather than a strictly enforced constraint. The feedback in this study did not include visual targets indicating successful or unsuccessful performance. The remaining fingers were flexed toward the palm. Participants received prior instructions and practiced each movement before the test. Tapping performance was evaluated using a magnetic sensor-based finger-tapping device, the UB-2. This device has been used to quantitatively assess finger function in various studies [9,25]. Magnets and magnetic sensors attached to the thumbs and index fingers detect changes in magnetic force during finger opening and closing. These signals are converted into electrical signals, from which the inter-finger distance is calculated. Motion parameters are then calculated using the UB-2 analysis algorithm. UB-2 calculates up to 248 parameters related to tapping speed and left-right coordination. Because this study examined the immediate effects of a motor feedback system, we limited the interpretation of short-term changes to outcome measures based on previous research. The selected indicators were (1) indicators reflecting finger-tapping speed and repetition, and (2) indicators reflecting left-right coordination. Table 1 summarizes the indicators used in this study.

### 2.4. Measurement Procedures

We compared finger-tapping performance with and without real-time visual finger-movement feedback. Each participant completed a total of four recorded 15 s trials, consisting of one trial under each feedback condition (feedback and no feedback) for each of the two task types (simultaneous and alternating tapping). All participants performed the simultaneous tapping task before the alternating tapping task, and the task order was fixed. Each trial lasted 15 s, and at least 1 min of rest was provided between consecutive trials.

To achieve an approximately balanced distribution of feedback-condition orders across the sample, participants were allocated to one of two order sequences based on the timing of participation. The first 20 participants (4 men and 16 women) performed the no-feedback condition followed by the feedback condition for both the simultaneous and alternating tapping tasks. The remaining 17 participants (3 men and 14 women) performed the feedback condition followed by the no-feedback condition for both task types. Thus, the feedback-condition order was approximately balanced across participants.

### 2.5. Data Analysis

The primary outcome measure was the number of taps, selected as the main indicator of repetitive finger-tapping performance. Secondary outcome measures included total travel distance, average phase difference between left- and right-hand tapping, standard deviation of phase difference, and similarity of hands, which characterize movement extent and bilateral coordination during the task. Statistical analyses were performed using parameter values automatically calculated for each participant by the UB-2 system.

For each task and feedback condition, descriptive statistics were calculated for each outcome (*n* = 37). Normality of the paired feedback differences (feedback—no feedback) was assessed using the Shapiro–Wilk test. Outcomes with normally distributed paired differences were presented as the mean ± standard deviation (SD), whereas outcomes with non-normally distributed paired differences were presented as the median (interquartile range).

To examine whether the effect of visual feedback on the primary outcome differed between the simultaneous and alternating finger-tapping tasks, a two-way repeated-measures analysis of variance was performed separately for each hand, with task (simultaneous and alternating) and feedback condition (no feedback and feedback) as within-participant factors. When the normality assumption for the interaction contrast was not met, the difference between the feedback and no-feedback conditions (feedback—no feedback) was calculated for each task and compared between the two tasks using the Wilcoxon signed-rank test as a sensitivity analysis.

The primary and secondary outcomes were also compared between the feedback and no-feedback conditions separately for each tapping task. For these task-specific comparisons, paired *t*-tests were used when the paired differences met the normality assumption, and Wilcoxon signed-rank tests were used otherwise. To examine potential feedback-condition order effects, change scores in the primary outcome (feedback—no feedback) were compared between the two feedback-order groups separately for each hand using Welch’s independent-samples *t*-test.

To account for multiple task-specific comparisons, Bonferroni-adjusted *p*-values were calculated within each outcome family. The adjustment included four comparisons for outcomes measured separately for each hand (two tasks × two hands) and two comparisons for bilateral coordination outcomes (two tasks). A Bonferroni-adjusted *p* < 0.05 was considered statistically significant.

Feedback effects were reported as mean paired differences with 95% confidence intervals. Cohen’s d_z and matched-pairs rank-biserial correlations (r_rb) were used as effect sizes for paired *t*-tests and Wilcoxon signed-rank tests, respectively. Statistical analyses were performed using IBM SPSS Statistics software (version 29.0; IBM Corp., Armonk, NY, USA).

## 3. Results

### 3.1. Effects of Visual Feedback on the Number of Taps

To examine whether the effect of visual feedback on the number of taps differed between the simultaneous and alternating finger-tapping tasks, a two-way repeated-measures analysis of variance was performed separately for each hand. For the nondominant hand, a significant main effect of task was observed (F(1,36) = 153.51, *p* < 0.001, partial η^2^ = 0.810), whereas the main effect of feedback was not significant (F(1,36) = 0.87, *p* = 0.357, partial η^2^ = 0.024). A significant Task × Feedback interaction was observed (F(1,36) = 4.30, *p* = 0.045, partial η^2^ = 0.107). The sensitivity analysis comparing the feedback effects (feedback—no feedback) between the two tasks also showed a significant difference (*p* = 0.030). For the dominant hand, a significant main effect of task was observed (F(1,36) = 159.99, *p* < 0.001, partial η^2^ = 0.816), whereas neither the main effect of feedback (F(1,36) = 1.10, *p* = 0.302, partial η^2^ = 0.030) nor the Task × Feedback interaction (F(1,36) = 2.73, *p* = 0.107, partial η^2^ = 0.071) was significant. The corresponding sensitivity analysis also showed no significant difference between the tasks (*p* = 0.110), although the change in the number of taps was greater during alternating tapping.

### 3.2. Alternating Finger-Tapping Task

Table 2 compares the no-feedback and real-time visual finger-movement feedback conditions for the alternating finger-tapping task. The number of taps was significantly higher in the feedback condition than in the no-feedback condition for both the nondominant and dominant hands after adjustment for multiple comparisons (adjusted *p* < 0.004 and *p* = 0.023, respectively). Total travel distance did not differ significantly between the feedback conditions for either hand. Since significant feedback-related differences in the number of taps were observed during alternating tapping, an additional analysis was conducted to examine potential order effects. The analysis of feedback-condition order showed no significant differences in change scores in the number of taps between the two feedback-order groups for either the nondominant hand (*p* = 0.278) or the dominant hand (*p* = 0.366).

### 3.3. Simultaneous Finger-Tapping Task

Table 3 compares the no-feedback and real-time visual finger-movement feedback conditions for the simultaneous finger-tapping task. The number of taps did not differ significantly between the two conditions for either the nondominant or dominant hand. Total travel distance was lower in the feedback condition than in the no-feedback condition for both hands; however, the difference was significant only for the dominant hand (adjusted *p* = 0.004) and not for the nondominant hand (adjusted *p* = 0.123).

### 3.4. Bilateral Coordination Outcomes

Table 4 presents the bilateral coordination results for the alternating and simultaneous finger-tapping tasks. The average phase difference, standard deviation of the phase difference, and similarity of hands did not differ significantly between the no-feedback and real-time visual finger-movement feedback conditions in either tapping task after adjustment for multiple comparisons.

## 4. Discussion

The primary finding of this study was that real-time visual feedback was associated with an increased number of taps during the more demanding alternating tapping task in both hands, whereas no significant increase was observed during simultaneous tapping. A significant Task × Feedback interaction was observed only in the nondominant hand. Rather than demonstrating a uniform benefit across tasks, these findings suggest that augmented visual feedback may support immediate motor performance in older adults, with its effects potentially varying according to task characteristics. To our knowledge, few studies have examined the task-dependent immediate effects of real-time visual feedback during finger tapping in older adults.

Presenting external feedback via visual or auditory channels may compensate for errors in self-perception of movement and promote action correction [31]. Because finger movements require dexterity and timing control, visual information about movement execution may facilitate online adjustments in motor performance. Accordingly, the real-time visual finger-movement feedback used in this study allowed participants to receive information about movement trajectory, rhythm, and coordination between the two hands. Participants may have used this information to make online adjustments, which may have contributed to the observed performance improvement in the alternating tapping task.

Regarding feedback effects among older adults, studies using knowledge of results (KR), which provides information about task outcomes after movement completion, have reported no significant effects [32]. Concurrent visual feedback showed improved task performance among older adults, although the improvement was less pronounced than among younger individuals [23]. Although motor abilities decline with age, performance improvements in motor learning remain achievable [17]. In this study, providing real-time feedback on movement information likely improved task performance, consistent with the findings of Yamamoto et al. [23]. Although the research questions and evaluation methods differed between the two studies, both studies suggest that visually presented information can support the motor performance of older adults. Intraindividual variability in tap intervals serves as an indicator of cognitive function [33], but most participants in this study exhibited high cognitive abilities with minimal variation. Although an increase in the number of taps was observed, it remains unclear whether cognitive factors contributed to this change. These findings suggest that external information may facilitate transient adaptations in motor performance among older adults and that such feedback may be useful for supporting immediate task performance.

Our previous study showed that even simple tapping movements among young adults benefited from feedback using this method [24]. Younger adults generally exhibit better proprioceptive function, including kinesthesia, than older adults [34]. This heightened proprioceptive ability may have helped younger participants detect and correct subtle movement errors in response to real-time feedback. Consequently, even during simple tapping movements, real-time feedback improved movement accuracy and enhanced performance among young adults. In contrast, in the present study of older adults, feedback-related improvements were observed primarily during alternating tapping. One possible explanation is that simultaneous tapping imposed lower timing demands than alternating tapping, leaving less room for measurable improvement. Age-related reductions in proprioceptive function may have made it more difficult for older adults to detect and correct subtle movement errors using real-time visual feedback. Additionally, during simultaneous tapping, total travel distance decreased significantly only in the dominant hand, whereas during alternating tapping, total travel distance did not change significantly and the number of taps increased in both hands. Because total travel distance reflects accumulated finger movement, its reduction during simultaneous tapping may reflect reduced movement amplitude rather than improved tapping performance. Taken together, these findings suggest that feedback effects may vary according to task characteristics, although the underlying mechanisms cannot be determined from the present study design. Therefore, further studies using tasks with varying difficulty levels and motor demands are required to clarify how real-time visual feedback influences finger movements.

Older adults require greater cognitive resources when performing motor tasks [35], and age-related decline in motor ability becomes more pronounced as task difficulty increases [36]. Feedback effects may be more pronounced in tasks requiring rhythm generation and timing adjustments, such as alternating tapping, than in bilateral synchronous tasks, such as simultaneous bilateral tapping. Conversely, it has been suggested that practice with highly difficult tasks fails to elicit neuroplasticity, potentially leading to a decline in skill retention [37]. Previous studies have also shown that individuals utilize feedback in different ways depending on the complexity of the task [38]. Similarly, in feedback interventions like the study reported here, the effects obtained may vary depending on the participants’ proficiency level in the movement. Additionally, older adults have been reported to rely more heavily on visual information for walking and postural control [39]. However, motor learning research suggests that excessive external feedback may induce feedback dependency and interfere with retention and transfer [40,41]. Therefore, when implementing feedback interventions for older adults, it is necessary to carefully consider the frequency and method of presentation.

The participants in this study may not represent the population that would benefit most from this intervention, as they exhibited relatively high cognitive and motor function. These findings may have limited generalizability because this study involved healthy older adults, the majority of whom were women. Regarding frail older adults and individuals with mild cognitive impairment or motor dysfunction, the greater potential for improvement in these populations may make immediate performance changes associated with feedback more observable. Therefore, the applicability of this system should be investigated in a broader range of participants. The changes observed in this study may reflect short-term changes in aspects of finger-tapping performance, particularly movement speed and repetitiveness, and may suggest an immediate effect of feedback during task execution. Although gaze direction was standardized in both conditions, the feedback condition necessarily involved viewing and processing dynamic movement-related information. Therefore, the observed effects may partly reflect differences in visual attention or motivation between the conditions, in addition to the effects of movement-related feedback. The absence of changes in coordination suggests that the observed effects may not be explained solely by increased effort; however, alternative interpretations, such as increased effort without accompanying improvements in motor ability, cannot be ruled out. The findings may reflect the influence of multiple factors, including visual feedback.

This study has several limitations. First, the present findings were limited to the immediate effects of feedback during finger-tapping tasks. Therefore, it remains unclear whether these effects generalize to other movements, translate into improvements in clinical dexterity, or persist after feedback removal. The clinical significance of the approximately 2–3 additional taps observed over 15 s also remains unclear. Future studies should examine retention and long-term effects using clinical dexterity assessments, including recently developed evaluation systems [42]. The optimal feedback design and duration for older adults should also be investigated. Second, because the order of the two task types was fixed, possible practice or fatigue effects across the tasks cannot be excluded. Participants may also have become more familiar with the visual feedback display during the earlier simultaneous tapping task. This increased familiarity could have partly contributed to the observed task-dependent feedback pattern. Furthermore, because each condition consisted of a single 15 s trial, trial-to-trial variability could not be assessed. Although the feedback-condition order was approximately balanced, possible practice, fatigue, carryover effects across conditions, movement amplitude, and visual information from the participants’ hands in the peripheral visual field may have partially affected performance. Additionally, in this experiment, because tapping width was not strictly controlled, we cannot completely rule out the possibility that it influenced performance. However, because the direction of change in total travel distance differed between the tasks, variations in tapping amplitude alone may not fully explain the observed pattern. Therefore, the present results do not necessarily reflect improvements in manual dexterity or motor control and should be interpreted with caution. Future studies should control movement amplitude, for example, by displaying a target range of motion on the feedback screen. Third, the sample size was relatively small, and the a priori sample-size calculation was based on detecting the within-participant effect of feedback rather than the Task Type × Feedback interaction. Consequently, adequate statistical power to detect the hypothesized interaction cannot be assured, and the non-significant interaction for the dominant hand should be interpreted cautiously. Future studies should determine the required sample size based on the expected interaction effect and confirm the present findings in a larger sample.

## 5. Conclusions

This study investigated whether a system providing feedback on finger movements during a finger-tapping task could improve tapping performance among older adults. Real-time visual finger-movement feedback was associated with an immediate increase in the number of taps during alternating tapping in both hands, whereas no significant change in the number of taps was detected during simultaneous tapping. A significant Task × Feedback interaction was observed only in the nondominant hand. Future studies should expand the target population and determine whether the observed changes translate into clinically meaningful improvements in manual dexterity and hand function and whether these effects persist over time. Addressing these issues will help clarify how real-time visual feedback can be most effectively applied in future finger-tapping interventions.

## Figures and Tables

**Table 1:** Parameters used in the analysis of finger tapping.

Outcome Measure	Description	Interpretation
Total travel distance (mm)	Refers to the total distance travelled by two fingers, regardless of whether they are opening or closing. It indicates the overall amount of movement.	Higher values indicate a greater cumulative amount of finger opening and closing movement, whereas lower values indicate a smaller amount.
Number of taps (taps)	The number of taps during the measurement period.	The higher the value, the better the indicator.
Avg phase difference between the left hand and right hand tapping (°)	Assuming a tap interval of 360°, this is the average of the time-angle values indicating the time difference between the left and right hands. Ideally, this value should be 0° for simultaneous two-hand tasks and 180° for alternating two-hand tasks.	For simultaneous taps, the closer the value is to 0°, the better the indicator; for alternating taps, the closer the value is to 180°, the better the indicator.
Standard deviation of phase difference between left- and right-hand tapping (°)	The standard deviation of the phase difference between left- and right-hand tapping, with one cycle of right-hand tapping defined as 360°.	The lower the value, the better the indicator.
Similarity of hands	An indicator of the similarity in finger-tapping movement patterns between the left and right hands. This value represents the maximum value of the cross-correlation function between the distance waveforms of the left and right hands.	For simultaneous taps, a value closer to 1 indicates higher performance. For alternating taps, a value farther from 1 indicates higher performance.

Abbreviations: Avg., average.

**Table 2:** Comparison of alternating finger-tapping performance in the dominant and non-dominant hands with and without feedback.

	Non-Dominant Hand					Dominant Hand				
Outcome	No Feedback	Feedback	Feedback Effect (95% CI)	Adjusted *p*	Effect Size	No Feedback	Feedback	Feedback Effect (95% CI)	Adjusted *p*	Effect Size
Number of taps (taps)	33 (28.5–36.5)	34 (31.0–40.0)	2.81 (1.05 to 4.57)	<0.004	rrb = 0.70	33.92 ± 9.58	36.54 ± 10.71	2.62 (0.81 to 4.44)	0.023	dz = 0.48
Total travel distance (mm)	4538.6 ± 1217.1	4821.7 ± 1484.7	283.10 (−11.68 to 577.89)	0.237	dz = 0.32	4763.5 ± 1136.2	4917.2 ± 1430.7	153.77 (−95.13 to 402.67)	0.873	dz = 0.21

Measurement results for alternating tapping using the UB-2. Values are presented as mean ± SD for outcomes analyzed using paired *t*-tests and median (IQR) for outcomes analyzed using Wilcoxon signed-rank tests. *p* values are Bonferroni-adjusted within each outcome family (four comparisons: two tasks × two hands). dz = Cohen’s dz; rrb = matched-pairs rank-biserial correlation; *n* = 37. Abbreviation: Avg., average.

**Table 3:** Comparison of simultaneous finger tapping in the dominant hand and non-dominant hand with and without feedback.

	Non-Dominant Hand					Dominant Hand				
Outcome	No Feedback	Feedback	Feedback Effect (95% CI)	Adjusted *p*	Effect Size	No Feedback	Feedback	Feedback Effect (95% CI)	Adjusted *p*	Effect Size
Number of taps (taps)	55 (43.5–63.5)	51 (44.0–64.5)	−0.92 (−4.39 to 2.55)	1.000	rrb = 0.03	55 (43.5–63.0)	52 (44.0–65.5)	−0.35 (−3.96 to 3.26)	1.000	rrb = 0.11
Total travel distance (mm)	4882.1 ± 1233.7	4603.49 ± 1355.2	−278.59 (−529.76 to −27.42)	0.123	dz = −0.37	5020.85 ± 1513.9	4594.9 ± 1573.6	−425.98 (−665.85 to −186.10)	<0.004	dz = −0.59

Measurement results for simultaneous tapping using the UB-2. Values are presented as mean ± SD for outcomes analyzed using paired *t*-tests and median (IQR) for outcomes analyzed using Wilcoxon signed-rank tests. *p* values are Bonferroni-adjusted within each outcome family (four comparisons: two tasks × two hands). dz = Cohen’s dz; rrb = matched-pairs rank-biserial correlation; *n* = 37. Abbreviation: Avg., average.

**Table 4:** Comparison of bilateral coordination in simultaneous and alternating tapping with and without feedback.

	Alternating Tapping					Simultaneous Tapping				
Outcome	No Feedback	Feedback	Feedback Effect (95% CI)	Adjusted *p*	Effect Size	No Feedback	Feedback	Feedback Effect (95% CI)	Adjusted *p*	Effect Size
Avg phase difference (°)	179.29 (167.92–199.66)	181.57 (163.89–196.88)	−1.64 (−7.92 to 4.63)	1.000	rrb = −0.06	−17.64 ± 32.02	−15.00 ± 32.69	2.65 (−5.00 to 10.29)	0.974	dz = 0.12
SD of phase difference (°)	34.30 ± 23.92	33.43 ± 22.63	−0.88 (−5.89 to 4.14)	1.000	dz = −0.06	27.27 ± 15.83	26.88 ± 16.84	−0.40 (−5.92 to 5.13)	1.000	dz = −0.02
Similarity of hands	0.15 ± 0.18	0.15 ± 0.17	−0.005 (−0.040 to 0.030)	1.000	dz = −0.05	0.92 (0.80–0.96)	0.90 (0.72–0.96)	−0.032 (−0.085 to 0.021)	0.449	rrb = −0.23

Measurement results for bilateral coordination outcomes during finger tapping using the UB-2. Values are presented as mean ± SD for outcomes analyzed using paired *t*-tests and median (IQR) for outcomes analyzed using Wilcoxon signed-rank tests. *p* values are Bonferroni-adjusted within each outcome family (two task-specific comparisons). dz = Cohen’s dz; rrb = matched-pairs rank-biserial correlation; *n* = 37. Abbreviation: Avg., average.

## Data Availability

The datasets used and analyzed in this study are available from the corresponding author upon reasonable request.

## Abbreviations

The following abbreviations are used in this manuscript:

ADLs	Activities of daily living
KR	Knowledge of results
